# Ska3 Ensures Timely Mitotic Progression by Interacting Directly With Microtubules and Ska1 Microtubule Binding Domain

**DOI:** 10.1038/srep34042

**Published:** 2016-09-26

**Authors:** Maria Alba Abad, Juan Zou, Bethan Medina-Pritchard, Erich A. Nigg, Juri Rappsilber, Anna Santamaria, A. Arockia Jeyaprakash

**Affiliations:** 1 Wellcome Trust Centre for Cell Biology, University of Edinburgh, Michael Swann Building, Max Born Crescent, Edinburgh, EH9 3BF UK; 2 Biozentrum, University of Basel, Klingelbergstrasse 50/70, CH-4056 Basel, Switzerland; 3 Chair of Bioanalytics, Institute of Biotechnology, Technische Universität Berlin, 13355 Berlin, Germany; 4 Cell Cycle and Cancer, Group of Biomedical Research in Gynaecology, Vall d’Hebron Research Institute, Barcelona, Spain

## Abstract

The establishment of physical attachment between the kinetochore and dynamic spindle microtubules, which undergo cycles of polymerization and depolymerization generating straight and curved microtubule structures, is essential for accurate chromosome segregation. The Ndc80 and Ska complexes are the major microtubule-binding factors of the kinetochore responsible for maintaining chromosome-microtubule coupling during chromosome segregation. We previously showed that the Ska1 subunit of the Ska complex binds dynamic microtubules using multiple contact sites in a mode that allows conformation-independent binding. Here, we show that the Ska3 subunit is required to modulate the microtubule binding capability of the Ska complex (i) by directly interacting with tubulin monomers and (ii) indirectly by interacting with tubulin contacting regions of Ska1 suggesting an allosteric regulation. Perturbing either the Ska3-microtubule interaction or the Ska3-Ska1 interactions negatively influences microtubule binding by the Ska complex *in vitro* and affects the timely onset of anaphase in cells. Thus, Ska3 employs additional modulatory elements within the Ska complex to ensure robust kinetochore-microtubule attachments and timely progression of mitosis.

Physical attachment of chromosomes to spindle microtubules, mediated by the kinetochore, is essential for accurately segregating chromosomes to the daughter cells during cell division^1^,^2^,^3^. The plus ends of the microtubules approaching the kinetochore undergo cycles of polymerization and depolymerization, a phenomenon known as dynamic instability, which results in a transient stabilization of ‘straight’ and ‘curved’ microtubule structures. The forces associated with microtubule dynamic instability are crucial to drive chromosome segregation^4^,^5^,^6^,^7^,^8^,^9^. Hence, to facilitate spindle-driven chromosome segregation kinetochores must remain stably attached to microtubules while these assume dynamic structures.

Among the kinetochore associated proteins, the four-subunit Ndc80 (Ndc80-Nuf2-Spc24-Spc25) and the three-subunit Ska (Ska1-Ska2-Ska3) complexes are the major microtubule binding factors responsible for maintaining load bearing kinetochore-microtubule attachments^10^,^11^,^12^,^13^,^14^. The Ndc80 complex is a ~55 nm long rod-shaped structure with microtubule- and kinetochore-binding globular domains (Ndc80-Nuf2 and Spc24-Spc25, respectively) separated by a long coiled coil^10^,^13^,^14^,^15^,^16^. Structural studies have suggested that the Ndc80 complex binds microtubules by recognizing α- and β- tubulin at both intra- and inter-tubulin dimer interfaces^14^. Consequently, the microtubule-binding ability of the Ndc80 complex is sensitive to microtubule conformations, ‘straight’ is preferred over ‘curved’^14^,^17^,^18^. Although the Ndc80 complex can also track depolymerizing microtubules when attached to microspheres^19^,^20^, the establishment of stable kinetochore-microtubule attachments in cells additionally requires the Ska complex^18^,^21^,^22^,^23^,^24^.

The Ska complex localizes at the outer kinetochore in an Ndc80-dependent manner and is regulated by Aurora B kinase^18^,^23^,^25^. Our previous structural work revealed that the human Ska complex is a ‘W’ shaped dimer of triple helical bundles (formed by Ska1, Ska2 and Ska3) with C-terminal domains of Ska1 and Ska3 sticking out at the ends of the dimer, and that the C-terminal domain of Ska1 is the major microtubule binding moiety of this complex^17^,^18^,^24^. The Ska1 C-terminal domain includes a winged-helix motif (amino acids 133–255, Ska1-MTBD), which, via multiple interaction sites, engages with surface-exposed regions of tubulin monomers. As these regions are not affected by microtubule polymerization/depolymerization, the Ska complex can bind dynamic microtubule structures indiscriminately. These observations suggested that the Ska complex likely plays a crucial role in allowing kinetochores to remain attached to the plus tips of dynamic spindle microtubules^17^,^18^. Like Ska1, the Ska3 C-terminal domain is also required for stabilizing kinetochore-microtubule attachments and correct mitotic progression^24^. However, despite the essential requirement of the Ska3 C-terminal domain for Ska complex functionality, the molecular mechanism by which Ska3 carries out its role remains largely unknown.

Here, we have combined biochemistry, Cross-Linking/Mass Spectrometry (CLMS), and siRNA rescue assays to characterize the Ska3 C-terminal domain and evaluate its impact on the microtubule binding properties of the Ska complex *in vitro* and *in vivo*. We show that the Ska3 C-terminal domain, which is predicted to be predominantly unstructured, contributes to the timely onset of anaphase by modulating the microtubule-binding ability of the Ska complex through direct interactions with both microtubules and Ska1-MTBD.

## Results and Discussion

### Ska3 possesses an intrinsically disordered C-terminal domain required for efficient microtubule binding of the Ska complex

**Figure 1:**
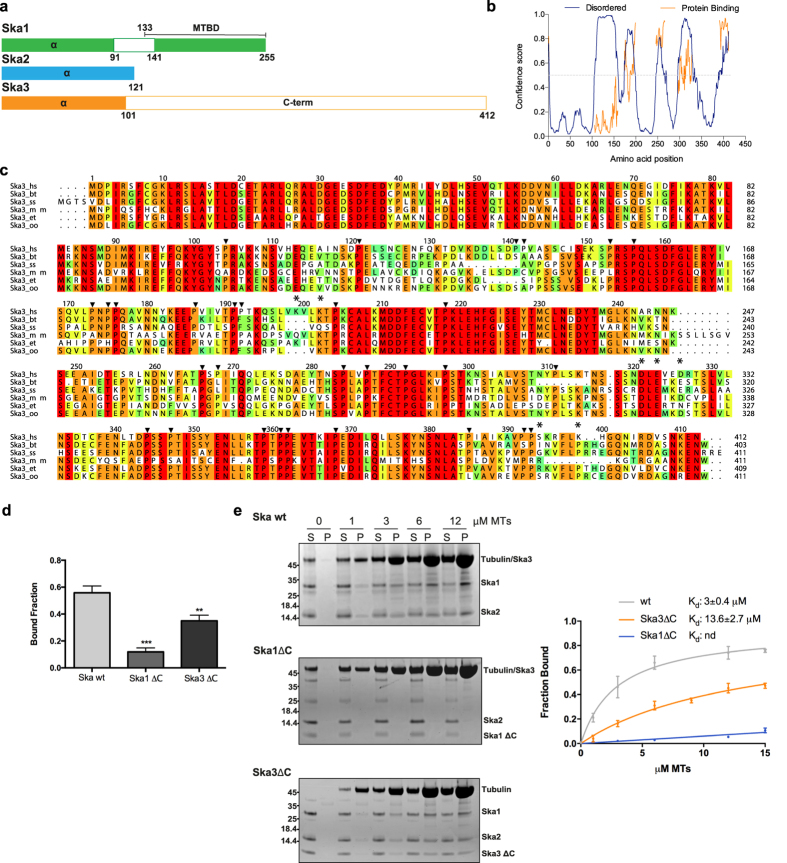
The intrinsically disordered C-terminal domain of Ska3 contributes to the microtubule binding activity of the Ska complex. (**a**) Domain architecture of the Ska components where filled boxes represent structured regions. MTBD: MicroTubule Binding Domain of Ska1 (residues from 133 to 255). (**b**) Predicted disordered and protein-binding regions of Ska3 using Disopred (http://bioinf.cs.ucl.ac.uk/psipred). Blue: predicted disordered residues, Orange: predicted protein-binding residues. (**c**) Amino acid conservation of Ska3 (conservation score is mapped from red to cyan, where red corresponds to highly conserved and cyan to poorly conserved). The alignments include orthologs from *H. sapiens* (hs), *Bos taurus* (bt), *Sus scrofa* (ss), *Mus musculus* (mm), *Echinops telfairi* (et), *Orcinus orca* (oo). Ska3 residues evaluated in this study are marked with asterisks. Multiple sequence alignment was performed with MUSCLE (MUltiple Sequence Comparison by Log-Expectation, EMBL-EBI) and edited with Aline^35^. (**d**) Quantification of MT-cosedimentation assay comparing the microtubule-binding activity of the wt Ska complex, Ska1ΔC and Ska3ΔC. Concentrations used in the assay: 1 μM protein, 9 μM MTs (mean ± s.d., n = 3, **P ≤
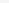
0.01, ***P ≤
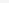
0.001; *t*-test). (**e**) Left, Representative SDS-PAGE of MT-cosedimentation assays comparing the microtubule-binding activity of the wt Ska complex, Ska1ΔC and Ska3ΔC. Right, Microtubule-binding curve for the wt Ska complex, Ska1ΔC and Ska3ΔC. K_d_ values were calculated using 1 μM Ska and 0–15 μM MTs (mean ± s.d., n = 4).

Amino acid sequence analysis of Ska3, using secondary structure and disorder prediction methods, revealed the presence of an intrinsically disordered C-terminal domain of unknown function (amino acids 102–412, Ska3_C-term_; Fig. 1a,b), with high propensity to participate in protein-protein interactions (Fig. 1b). This domain is highly acidic in nature (theoretical isoelectric point is ~4.5) and possesses a high proportion of prolines (30 out of 311 residues; 9.6%), features that are remarkably conserved among species (Fig. 1c). Our previous work showed that the Ska3_C-term_, although did not bind microtubules on its own, when removed from the Ska complex reduced the microtubule ability of the latter^24^. This had suggested that Ska3_C-term_ might favorably contribute to the microtubule binding of the Ska complex by creating microtubule-binding interfaces, together with Ska1, when connected to the structural core of the Ska complex made of coiled-coils^24^. To quantitatively assess this possibility, we carried out microtubule cosedimentation assays with Ska complexes lacking the C-terminal domain of Ska1 (Ska1_1–91_-Ska2-Ska3; Ska1ΔC) or the C-terminal domain of Ska3 (Ska1-Ska2-Ska3_1–101_; Ska3ΔC). While the Ska3ΔC complex showed reduced microtubule binding, the Ska1ΔC complex, as expected^24^, failed to interact with microtubules (Fig. 1d and e, Supplementary Fig. S1a). Microtubule binding affinity of the Ska3ΔC complex, as measured by the microtubule cosedimentation assay, is 13.6 μM, which is 4.5 fold lower than that of wild type (wt) Ska complex comprising full length Ska3 (Kd = 3 μM) (Fig. 1e). The analysis of the measured molecular mass of wt Ska complex and Ska3ΔC, determined by SEC-MALS (Size Exclusion Chromatography combined with Multi-Angle Light Scattering), showed that the removal of the Ska3_C-term_ did not alter the oligomeric state of the complex (Supplementary Fig. S1b). Therefore, the reduced microtubule-binding of the Ska3ΔC complex is a direct consequence of the loss of the C-terminal domain of Ska3.

**Figure 2:**
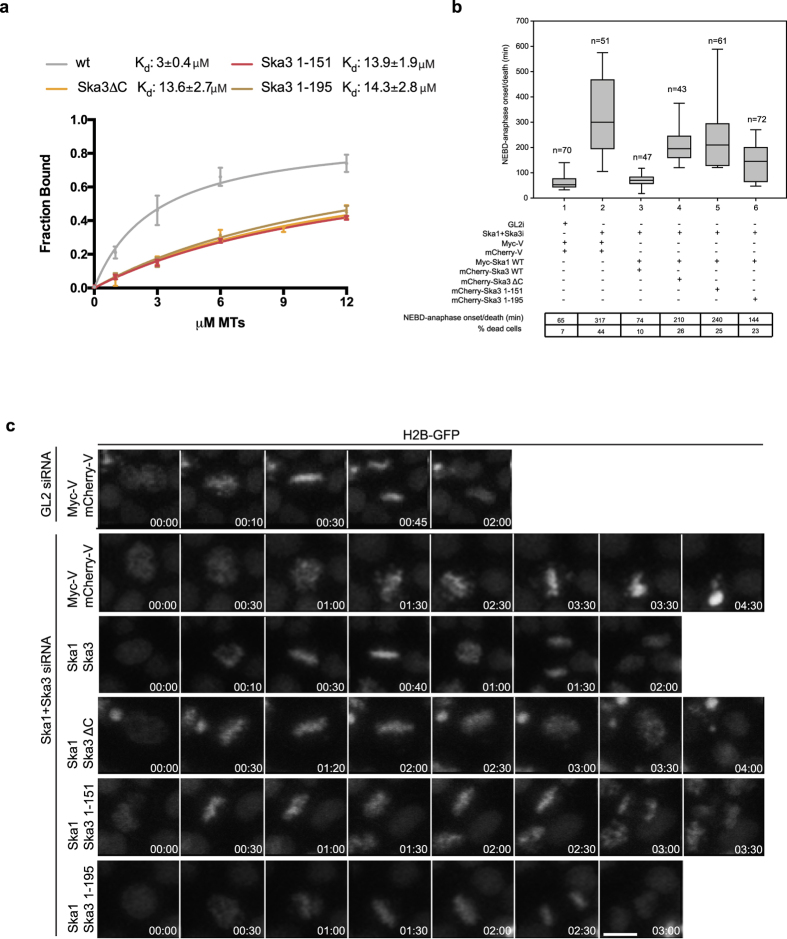
The C-terminal domain of Ska3 is required for timely mitotic progression. (**a**) Quantification of MT-cosedimentation assays comparing the wt Ska complex and Ska1-Ska2-Ska3ΔC with Ska1-Ska2-Ska3_1–151_ and Ska1-Ska2-Ska3_1–195_. K_d_ values were calculated using 1 μM Ska and 0–12 μM MTs (mean ± s.d., n = 4). (**b**) Box-and-whisker plot showing the elapsed time (min) between nuclear envelope breakdown (NEBD) and anaphase onset/death for individual cells. The number of cells (n) from three independent time-lapse experiments is given above each box. Lower and upper whiskers represent 10^th^ and 90^th^ percentiles, respectively. Below, table summarizing information from the live cell experiments regarding the average time between NEBD to anaphase onset/death and the percentage of cells dying in mitosis. Myc-V and mCherry-V were used as transfection controls. (**c**) Representative stills from time-lapse video-microscopy experiments illustrating mitotic progression of HeLa S3 cells stably expressing histone H2B-GFP treated as in (**b**). Time in hr:min is indicated. T = 0 was defined as the time point where NEBD became evident. Scale bar, 10 μm.

To narrow down the region of Ska3 that influences the microtubule binding properties of the Ska complex, we reconstituted Ska complexes containing two different C-terminal truncations of Ska3 that were designed based on the presence of conserved amino acid stretches (Ska1-Ska2-Ska3_1–151_ ; Ska1-Ska2-Ska3_1–195_). As expected, SEC-MALS analyses confirmed that the truncation of Ska3 C-terminal regions does not affect the overall oligomeric structure of these complexes (Supplementary Fig. S2a). Microtubule cosedimentation assays showed that these complexes bound microtubules with affinities similar to that of Ska1-Ska2-Ska3ΔC (Fig. 2a, Supplementary Fig. S2b; Kd = 13.9 μM and 14.3 μM, respectively), indicating that the Ska3 region between residues 196 and the C-terminal end is critical in modulating the microtubule binding of the Ska complex.

Considering the modest contribution of Ska3_C-term_ for microtubule binding *in vitro*, we assumed that any siRNA-based rescue evaluation of this domain *in vivo* needs to be carried out in cells where complete depletion of the Ska complex is achieved (to avoid stabilization of remaining endogenous complex that could mask the specific Ska3_C-term_ contribution). We previously showed that co-depletion of Ska1 and Ska3 led to an efficient reduction of the endogenous Ska complex^23^,^24^ (Supplementary Fig. S2c). Hence, we first co-depleted Ska1 and Ska3 in HeLa S3 cells stably expressing H2B-GFP and simultaneously co-transfected full length Myc-Ska1 and the different truncations of Ska3 tested *in vitro*, namely mCherry-Ska3_1–101_, Ska3_1–151_ or Ska3_1–195_ (representing Ska1-Ska2-Ska3ΔC, Ska1-Ska2-Ska3_1–151_ and Ska1-Ska2-Ska3_1–195_ complexes, respectively). The contribution of different Ska3 C-terminal regions was assessed by quantifying the timing between nuclear envelope breakdown (NEBD) and anaphase onset, taken as the time when sister chromatids separate from each other. Cells reconstituted with Ska1-Ska2-Ska3 complex (containing Myc-Ska1 and mCherry-Ska3) progressed through mitosis similarly to the control (GL2-treated, siRNA against firefly luciferase) cells. Consistent with the *in vitro* data (Fig. 2a, as well as our previous observations in the case of Ska1-Ska2-Ska3ΔC^24^), expression of Ska complexes comprising C-terminally truncated Ska3 resulted in a prolonged delay in anaphase onset and an increase in the number of dead cells (Fig. 2b,c). These observations demonstrate the role of Ska3_C-term_ in modulating the microtubule binding ability and function of the Ska complex. Noticeably, Ska1-Ska2-Ska3ΔC and Ska1-Ska2-Ska3_1–151_ exhibited a slightly stronger phenotype (~3 fold delay in anaphase onset) as compared to Ska1-Ska2-Ska3_1–195_ (2 fold delay). In light of the data shown in Fig. 2a, this suggests a microtubule-binding independent role for the Ska3 region spanning residues 152–194.

### Ska3 C-terminal domain directly interacts with microtubules and Ska1-MTBD

**Figure 3:**
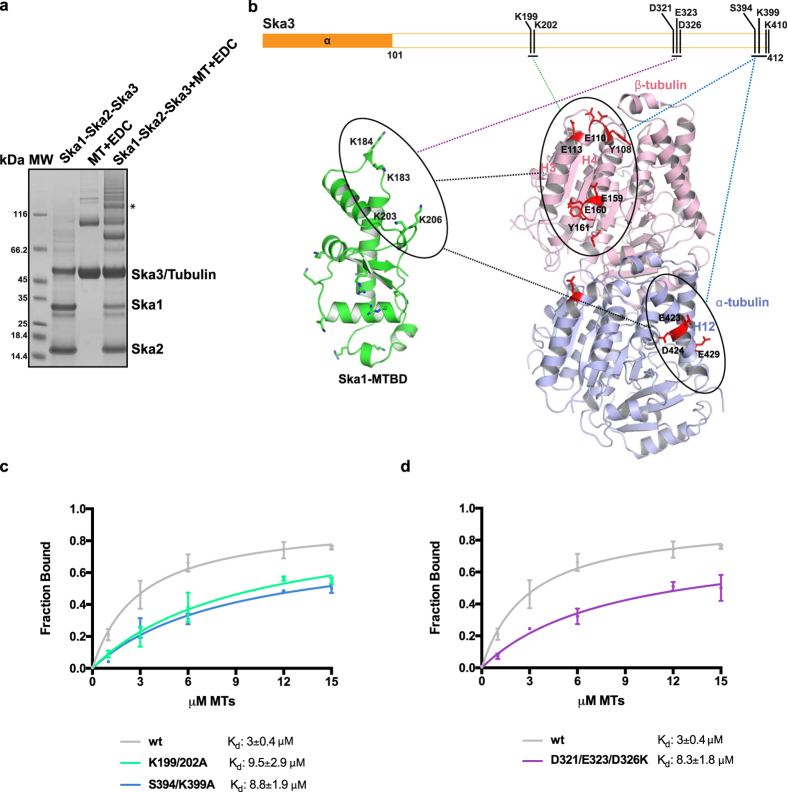
Ska3 directly interacts with tubulin monomers and Ska1-MTBD. (**a**) Representative SDS-PAGE of 6 μM Ska complex cross-linked to 10 μM microtubules with EDC. The band analyzed in this study is marked with an asterisk. (**b**) Cartoon representation of tubulin dimer (pink: β-tubulin, blue: α-tubulin) where residues involved in cross-linking with Ska3 (orange) and Ska1-MTBD (green) are shown in stick representation. Green, blue and purple lines indicate cross-links observed between Ska3 K199/K202 and β-tubulin, Ska3 S394/K399 and α- and β- tubulin, and Ska3 D321/E323/D326 and Ska1, respectively. (**c**) Microtubule-binding curves of the wt Ska complex (grey) and Ska3 microtubule-binding mutants (K199/202A and S394/K399A; green and blue respectively). K_d_ values were calculated using 1 μM Ska and 0–15 μM MTs (mean ± s.d., n ≥ 4). (**d**) Microtubule-binding curve of the wt Ska complex (grey) and Ska1-binding inefficient Ska3 mutant (D321/E323/D326K, purple). K_d_ values were calculated using 1 μM Ska and 0–15 μM MTs (mean ± s.d., n ≥ 4).

To better understand the molecular basis for the contribution of Ska3_C-term_ microtubule binding, we re-examined the CLMS experiments of our previous study^17^, which used a zero-length cross-linker EDC (1-ethyl-3-[3-dimethylaminopropyl] carbodiimide hydrochloride) and dissected the Ska1-microtubule binding interface through the identification of specific intermolecular crosslinks involving Lys (and less favorably Ser, Thr and Tyr) and Asp/Glu. We have now analysed the higher molecular weight cross-linked products containing all the subunits of the Ska complex and microtubules. This revealed cross-links involving the Ska3_C-term_ residues: (i) Lys199/Lys202/Ser394/Lys399/Lys410 and β tubulin; (ii) Lys399 and α tubulin and (iii) Asp321/Glu323/Asp326 and Ska1-MTBD residue Lys183, part of a Lys cluster (Lys183/Lys184/Lys203/Lys206) previously identified as important for microtubule binding^17^ (Fig. 3a,b, Supplementary Fig. S3a). We note that the Ska3 residues identified by CLMS are either conserved or flanked by highly conserved regions (Fig. 1c). Interestingly, Ska3_C-term_ contacts tubulin monomers at the same sites where we had previously shown Ska1-MTBD to engage with microtubules (H3 and H4 of β-tubulin and H12 of α-tubulin) (Fig. 3b). These observations suggested that Ska3_C-term_ contributes to the Ska complex microtubule binding by directly contacting both the tubulin monomers and Ska1-MTBD. To validate this, we mutated the Ska3_C-term_ residues contacting microtubules (Ska3 K199/202A and Ska3 S394/K399A) and Ska1-MTBD (Ska3 D321/E323/D326K) and tested them separately in microtubule cosedimentation assays. Importantly, all Ska complexes containing the above mentioned Ska3 mutants bound microtubules less efficiently as compared to the wt Ska complex (Fig. 3c,d, Supplementary Fig. S3b). The size exclusion chromatography profiles of the Ska3 mutant Ska complexes were almost identical (Supplementary Fig. S3c), suggesting that the mutations do not perturb the overall architecture of the Ska complex. Thus, we conclude that the effects of these mutations on microtubule binding activity are due to the perturbation of Ska3_C-term_-microtubule and Ska3_C-term_-Ska1-MTBD interactions.

### Ska3_C-term_ microtubule binding and Ska1-MTBD binding activities are required for timely onset of anaphase

**Figure 4:**
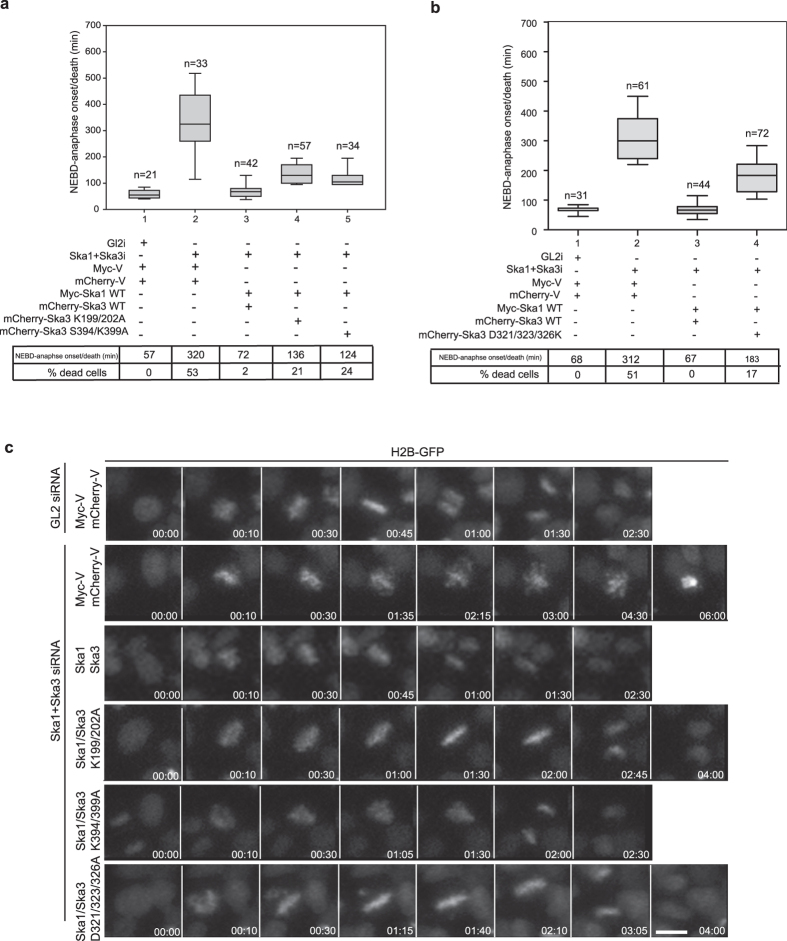
Direct binding of Ska3_C-term_ to Ska1 and microtubules is required for maintaining kinetochore-microtubule attachments and timely anaphase onset. (**a**,**b**) Box-and-whisker plot showing the elapsed time (min) between nuclear envelope breakdown (NEBD) and anaphase onset/death for individual cells. The total number of cells (n) from three independent experiments is given above each box. Lower and upper whiskers represent 10^th^ and 90^th^ percentiles, respectively. Table summarizing information from the live cell experiments shown below regarding the average time between NEBD to anaphase onset/death and the percentage of cells dying in mitosis. (**c**) Representative stills from time-lapse video microscopy experiments illustrating mitotic progression of HeLa S3 cells stably expressing H2B-GFP, treated as in (**a,b**). Time in hr:min is indicated. T = 0 was defined as the time point at which NEBD became evident. Scale bar, 10 μm.

We next evaluated the consequences of disrupting Ska3_C-term_–microtubule and Ska3_C-term_-Ska1-MTBD interactions on mitotic progression, using siRNA rescue assays with the Ska3 mutants characterized above. In agreement with the *in vitro* results, rescues by the Ska3 K199/202A and K394/399A resulted in perturbed mitotic progression, characterized by about a 2 fold delay in anaphase onset and a significant increase in the number of dead cells (Fig. 4a,c). Similarly, the Ska3 D321/323/326K mutant, which is predicted not to interact with Ska1-MTBD, resulted in a 3 fold delay in anaphase onset with around 17% cells undergoing cell death (Fig. 4b,c), highlighting the requirement for Ska3_C-term_–microtubule and Ska3_C-term_-Ska1-MTBD interactions. All Ska3 mutants tested were able to form a complex with the other Ska subunits comparable to wt Ska3 (Supplementary Fig. S4; shown for Ska1), indicating that the observed mitotic defects are not due to disruption of complex formation. We also noted that the majority of cells expressing these mutants were able to align their chromosomes in a timely manner (data not shown), as seen for the Ska1 microtubule binding deficient mutants^17^, suggesting that the Ska3_C-term_ contributes to the robustness of kinetochore-microtubule attachments, rather than to the establishment of the initial kinetochore-microtubule contacts.

Overall this study identifies the microtubule and Ska1-contacting regions of Ska3_C-term_ and shows that these intermolecular contacts act as important modulatory elements within the Ska complex to ensure timely mitotic progression. The Ska3_C-term_, although predicted to be unstructured, possesses highly conserved amino acid stretches likely to be involved in protein-protein interactions, and this suggests an evolutionarily conserved role of this domain for Ska complex function and error free cell division. What other factors of the kinetochore the Ska3_C-term_ binds to and how microtubule-binding factors of the Ska complex cooperate with the Ndc80 complex are important open questions to understand the mechanistic details of spindle driven chromosome segregation.

## Methods

### Recombinant protein purification and microtubule cosedimentation assay

Ska1, Ska2 and Ska3 V58I were cloned individually in a pEC-S-CDF-His, pEC-A-HT-His-GST, pEC-K-HT-His vector respectively with TEV cleavage sites. Ska3 mutants and truncations were generated following the Quikchange site-directed mutagenesis method (Stratagene). All protein complexes were expressed in *E. coli* strain BL21 Gold by co-transforming all the three plasmids containing individual Ska components. Protein expressions were induced overnight at 18 ^o^C and purified using the same protocol as described before. Briefly, after cell lysis in 20 mM Tris pH 8, 500 mM NaCl and 5 mM DTT, the protein complexes were purified by affinity chromatography in batch mode using glutathione sepharose (GE healthcare) beads and thoroughly washed with 20 mM Tris pH 8, 500 mM NaCl and 5 mM DTT, followed by 20 mM Tris pH 8, 1 M NaCl, 50 mM KCl, 10 mM MgCl_2_, 2 mM ATP and 5 mM DTT then finally with 20 mM Tris pH 8, 100 mM NaCl and 5 mM DTT. The proteins were eluted with 50 mM glutathione, 20 mM Tris pH 8, 100 mM NaCl and 5 mM DTT and the tags were cleaved with TEV protease overnight. Final size-exclusion chromatography step was carried out in 20 mM Tris-HCl pH 8, 100 mM NaCl and 5 mM DTT (Superose 6, GE Healthcare).

Microtubule cosedimentation assays were performed as previously described^17^. Tubulin was purchased from Cytoskeleton Inc. and microtubules were polymerized according to manufacturer’s instructions. Taxol-stabilized microtubules were incubated at room temperature for 10 min with 1 μM protein in a 50 μl reaction volume in BRB80 buffer (80 mM PIPES pH 6.9, 1 mM EGTA, 1 mM MgCl2) with 100 mM NaCl and 4 mM DTT in the presence of 50 μM taxol. The reaction was then layered onto a 250 μl glycerol cushion buffer (BRB80, 50% Glycerol, 4 mM DTT and 50 μM taxol) and ultracentrifuged for 10 min at 80,000 rpm in a Beckman TLA 100.3 rotor at 25 °C. Pellets and supernatants were analyzed by SDS-PAGE, stained with Coomassie Blue and quantification was performed with ImageJ. Normalized binding data were obtained by dividing the band intensity of the pellet fraction by the sum of pellet and supernatant band intensities. The K_d_ (concentration needed to achieve half-maximal binding) was determined by nonlinear regression using GraphPad Prism (GraphPad Software, La Jolla California, USA).

### Chemical Cross-linking/Mass Spectrometry (CLMS)

Cross-linking experiments were carried out using EDC (Thermo Fisher Scientific) in the presence of N-hydroxysulfosuccinimide (Sulfo-NHS, Thermo Fisher Scientific) as described before^17^. In brief, the gel bands of cross-linked complexes were processed following standard procedures^26^ and digested peptides were desalted using C18 StageTips^27^,^28^. Peptides were analysed on an LTQ Orbitrap Velos mass spectrometer (Thermo Fisher Scientific) that was coupled with a Dionex Ultimate 3000 RSLC nano HPLC system, using a high/high strategy^29^: both MS spectra and MS2 spectra were acquired in the Orbitrap. The mass spectrometric raw data were processed to generate peak lists by MaxQuant (Version 1.5.1.2)^30^ and cross-linked peptides were matched to spectra using Xi software (ERI, Edinburgh). The mass spectrometry proteomics data have been deposited to the ProteomeXchange Consortium via the PRIDE^31^ partner repository with the dataset identifier PXD004952.

### Cell culture and siRNA rescue experiments

HeLa S3 cells expressing histone H2B-GFP^32^ were maintained in DMEM (Invitrogen) supplemented with 10% fetal bovine serum and penicillin/streptomycin (100 IU ml^−1^ and 100 mg ml^−1^, respectively; Gibco). siRNA rescue experiments were carried out as previously described^24^. siRNA-mediated depletion of Ska1 and Ska3 was carried out during 72 h and the siRNA oligonucleotides were transfected using Oligofectamine (Invitrogen) according to manufacturer’s instructions. The sequences of Ska1, Ska3 and control GL2 duplexes are: 5′-CCCGCTTAACCTATAATCAAA-3′, 5′-AGACAAACATGAACATTAA-3′ and 5′-AACGTACGCGGAATACTTCGA-3′, respectively^17^,^21^,^23^,^24^,^33^. Ska1 and Ska3 siRNA-resistant constructs were generated in the pcDNA3.1 plasmid (Invitrogen), carrying a N-terminal triple-Myc tag (in the case of Myc-Ska1) or a single mCherry tag (in the case of Ska3). Myc-V and mCherry-V were used as transfection controls. Plasmids were transfected using X-tremeGENE HP (Roche) according to manufacturer’s instructions. For synchronization studies, cells were arrested for 20 h with 2 mM thymidine, followed by a release into fresh medium for 6–8 h and a second thymidine block of 16 h and release for 10 h before fixation or visualization.

### Time-lapse microscopy

For time-lapse microscopy, all treatments within a single experiment were performed simultaneously. Cells stably expressing H2B-GFP and transiently expressing the corresponding mCherry constructs were imaged using an Olympus multidimensional microscope CellR IX81 equipped with a 150 W MT-ARC/XeHg excitation system, a 20x/NA 0.45 and a C9100-02 EMCCD camera. During imaging the atmosphere was maintained at a temperature of 37 °C and 5% CO_2_. Images were captured at 5-min intervals for 22 h at multiple positions. GFP and mCherry fluorescence images were acquired with 30 ms and 60 ms exposure times, respectively. mCherry fluorescence was imaged only every five time points to monitor transfected cells. Time taken for the transition from nuclear envelope breakdown (NEBD) to anaphase onset (taken as the time when sister chromatids separate from each other) was quantified and ImageJ software was used to process the data.

### Immunoprecipitation and Western blotting

For immunoprecipitations, lysates (1–2 mg total protein amount) from HEK293T cells were incubated for 2 h at 4 °C with 9E10 anti-Myc antibody. In each case, 1 mg of antibody was coupled to 1 ml sepharose-A beads (Pierce, Rockford, IL). After protein capture, beads were washed 4x with HEPES lysis buffer (50 mM Hepes pH 7.4, 150 mM NaCl, 0.5% Triton X-100, 30 μg/ml RNase, 30 μg/ml DNase, 100 μM DTT, protease inhibitors (ROCHE), cocktail of phosphatase inhibitors (Sigma)) and resuspended in gel sample buffer and proteins were analyzed by SDS–PAGE and immunoblotting. Membranes were probed with the following antibodies: rabbit anti-mCherry^17^ (1:3000), mouse anti-myc (clone 9E10)^34^ (1:10), anti-Ska1 (1:1000)^21^, anti-Ska3 (1:3000)^23^ and mouse anti-tubulin antibody (Sigma-Aldrich) (1:1000) as reported previously^21^.

## Additional Information

**How to cite this article**: Abad, M. A. *et al.* Ska3 Ensures Timely Mitotic Progression by Interacting Directly With Microtubules and Ska1 Microtubule Binding Domain. *Sci. Rep.* **6**, 34042; doi: 10.1038/srep34042 (2016).

## Supplementary Material

Supplementary Information
