# Supplementary material for: Ska3 Ensures Timely Mitotic Progression by Interacting Directly With Microtubules and Ska1 Microtubule Binding Domain
Source: Sci Rep. 2016 Sep 26;6:34042. doi: 10.1038/srep34042 (PMC5036024; doi:10.1038/srep34042)
Supplement: Supplementary Information [file srep34042-s1.pdf]

Ska3 Ensures Timely Mitotic Progression by Interacting Directly With Microtubules and Ska1 Microtubule Binding Domain

Maria Alba Abad[1], Juan Zou[1], Bethan Medina-Pritchard[1], Erich, A. Nigg[2], Juri Rappsilber[1], Anna Santamaria[3] and A. Arockia Jeyaprakash[1]*.

[1]*Wellcome Trust Centre for Cell Biology, University of Edinburgh, Michael Swann Building, Max Born Crescent, Edinburgh, EH9 3BF*
[2]*Biozentrum, University of Basel, Klingelbergstrasse 50/70, CH-4056 Basel, Switzerland*
[3]*Cell Cycle and Cancer, Group of Biomedical Research in Gynecology, Vall d'Hebron Research Institute, Barcelona, Spain*

Correspondence: ajeyapra@staffmail.ed.ac.uk

Running title: Ska3-microtubule interactions

a

b

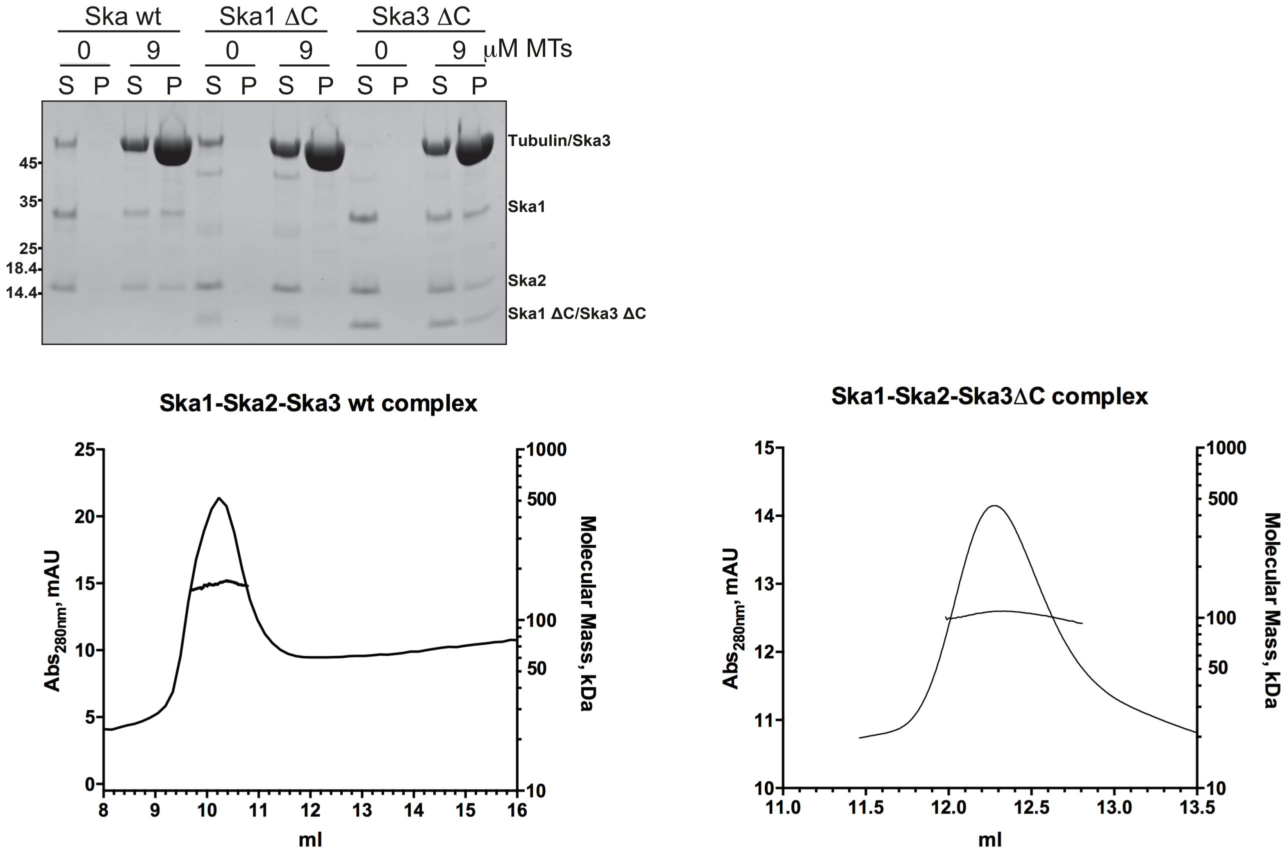

	Measured MW in kDa (Calculated MW)	Quaternary Structure
Ska1-Ska2-Ska3	169kDa (180KDa)	Homodimer
Ska1-Ska2-SKa3ΔC	121kDa (112kDa)	Homodimer

Figure S1. **a**) Representative SDS-PAGE gels of cosedimentation assays comparing the microtubule binding ability of the wt Ska complex, Ska1ΔC and Ska3ΔC as shown in **Fig. 1d**. **b**) SEC-MALS profile of the wt Ska complex (left) and Ska3ΔC (right). The analysis shows that the deletion of the C-terminal domain of Ska3 does not alter the overall oligomeric state of the complex.

a

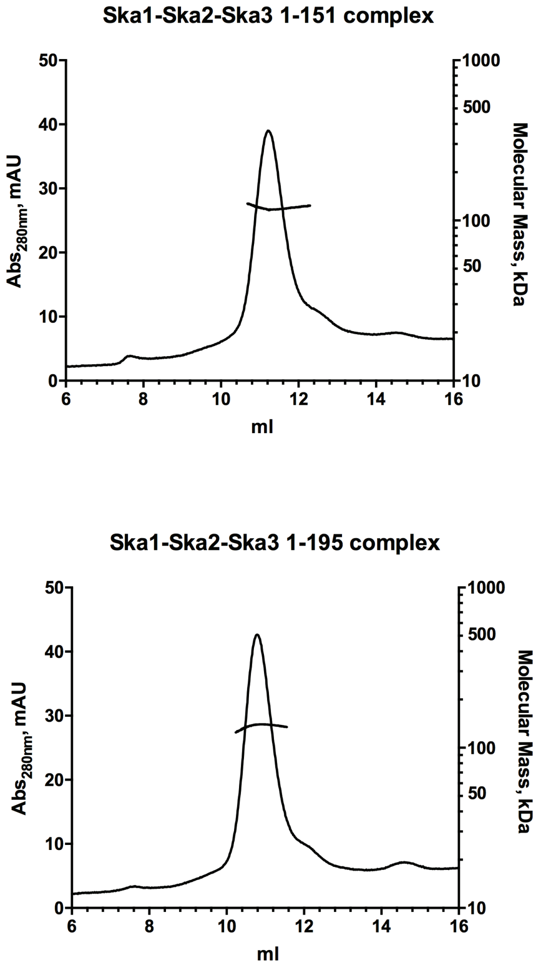

	Measured MW in kDa (Calculated MW)	Quaternary Structure
Ska1-Ska2-Ska3 1-151	121.8kDa (122KDa)	Homodimer
Ska1-Ska2-SKa3 1-195	132.9kDa (132kDa)	Homodimer

b

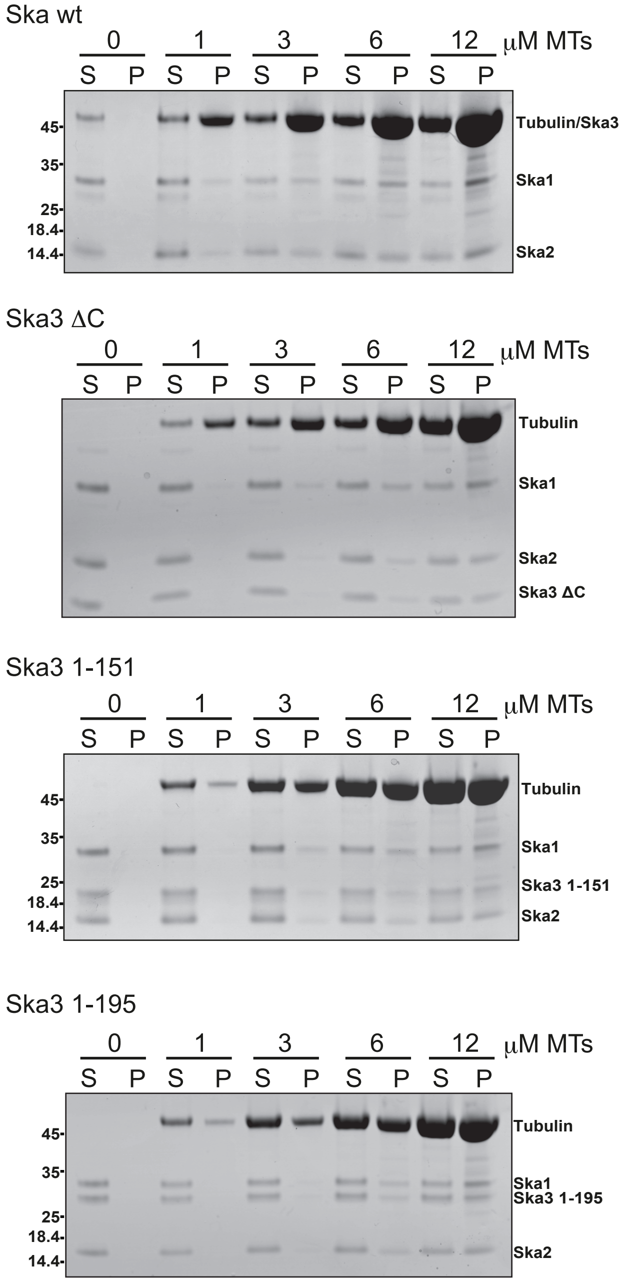

c

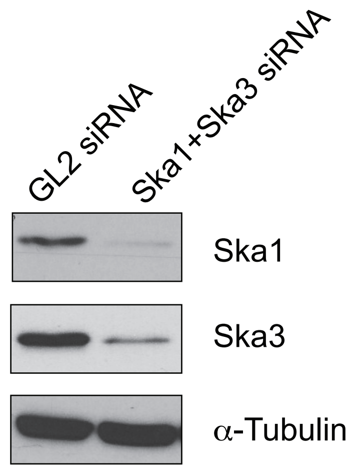

Figure S2. **a)** SEC-MALS profiles of the Ska1-Ska2-Ska3 1-151 (top) and Ska1-Ska2-Ska3 1-195 (bottom) complexes. The analysis shows that the truncations do not affect the oligomeric structure of the complex. **b)** Representative SDS-PAGE gels of cosedimentation assays comparing wt Ska complex and Ska3ΔC with Ska1-Ska2-Ska3 1-151 and Ska1-Ska2-Ska3 1-195 as shown in **Fig. 2a**. **c)** Representative western blot showing the level of depletion obtained in HEK293T with Ska1 and Ska3 double siRNA after 72 hours of treatment.

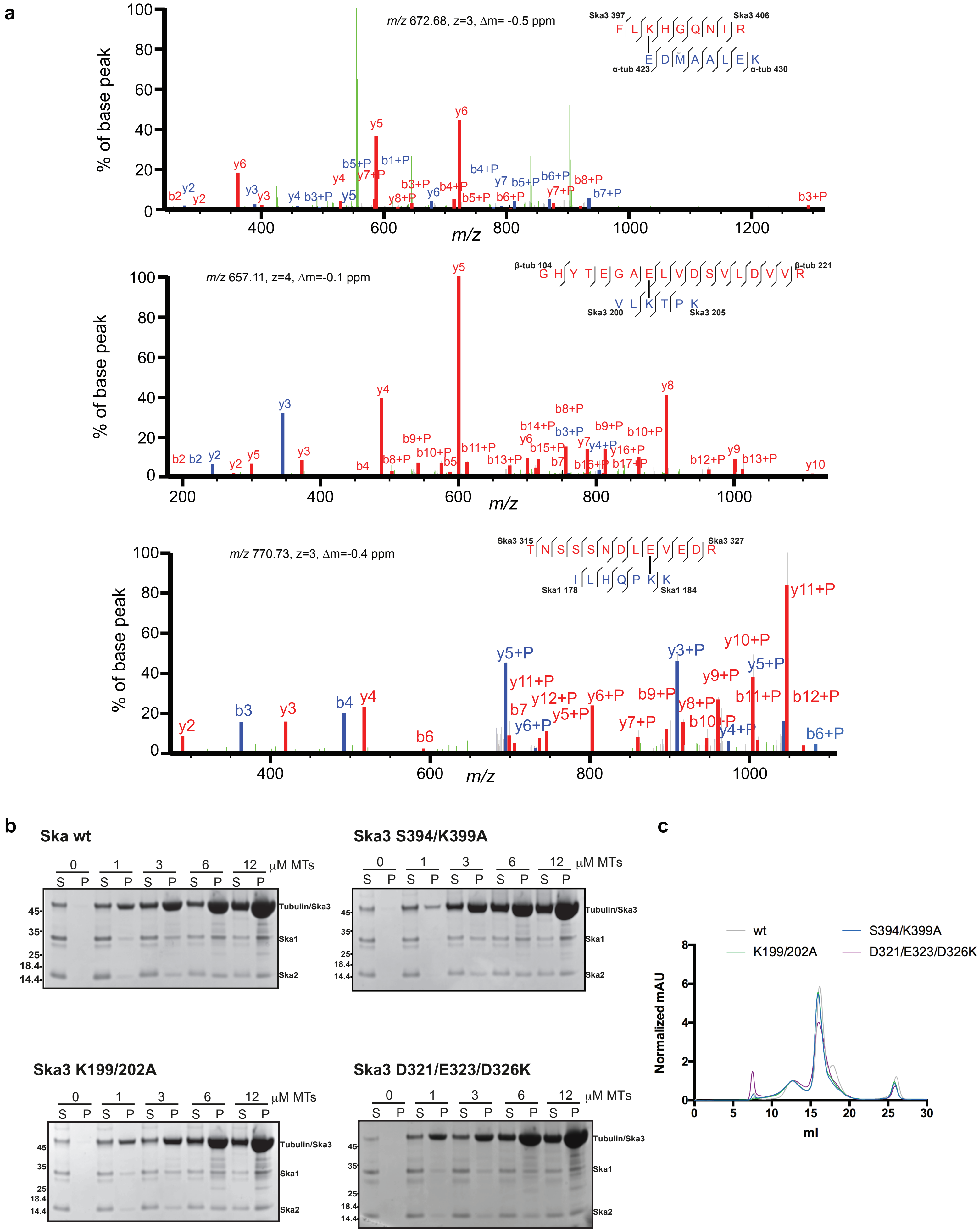

Figure S3. **a**) High resolution fragmentation spectra of a few representative cross-linked peptides. **b**) Representative SDS-PAGE gels of MT-cosedimentation assays comparing wt Ska complex and Ska3 point mutants (Ska3 K199/202A, S394/K399A and D321/E323/D326K) as shown in **Fig. 3c** and **d**. **c**) Comparison of the size exclusion chromatograms of all the mutants tested in MT-cosedimentation assays shown in **Fig. 3c** and **d**. Analysis shows that all mutants behave identically to the wt Ska complex.

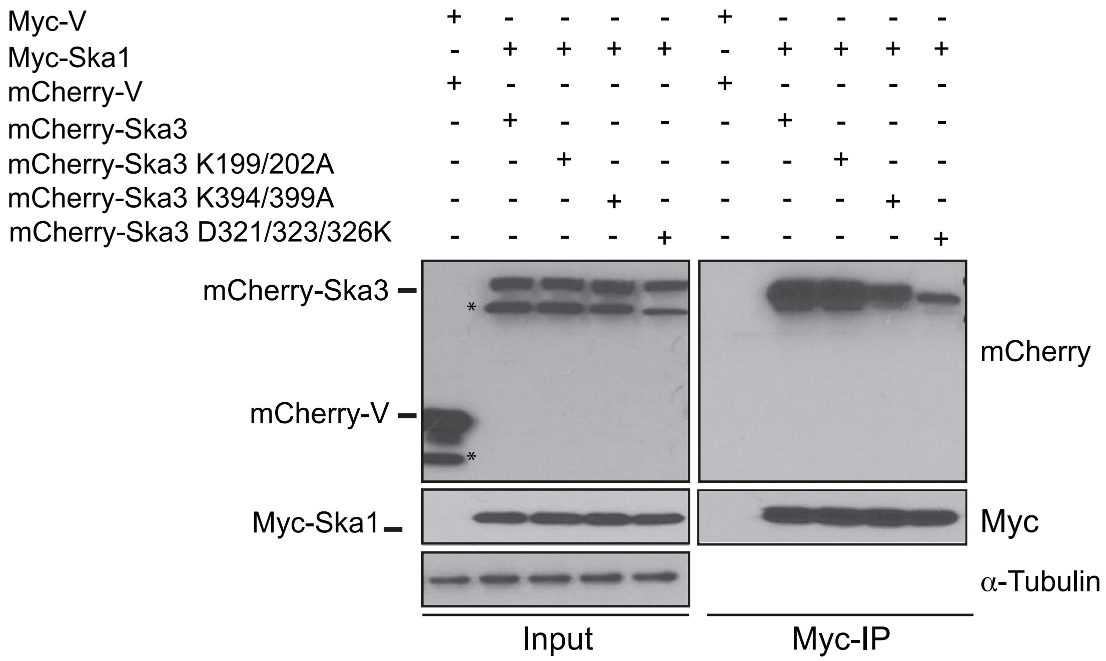

Figure S4. Combinations of Myc-Ska1 and mCherry-Ska3 versions as depicted in the figure were expressed in HEK-293T cells and cells were harvested after 36 hours. Myc-empty vector or Myc-Ska1 was then immunoprecipitated using anti-Myc antibodies, resolved by SDS-PAGE and co-immunoprecipitation of mCherry-Ska3 versions was resolved by Western blotting.
